# Supplementary material for: Multilevel analysis of dropout from maternal continuum of care and its associated factors: Evidence from 2022 Tanzania Demographic and Health Survey
Source: PLoS One. 2024 May 7;19(5):e0302966. doi: 10.1371/journal.pone.0302966 (PMC11075823; doi:10.1371/journal.pone.0302966)
Supplement: S1 Table — (DOCX) [file pone.0302966.s001.docx]

S1 Table. Background characteristics of respondents and distribution of dropout from maternity CoC across independent variables among reproductive-age women in Tanzania (n=5, 172)

| **Variable name** | **Categories** | **ANC** | | **Institutional delivery** | | **Postnatal care visit** | |
| --- | --- | --- | --- | --- | --- | --- | --- |
|  |  | **1-3** | **≥4** | **Home delivery** | **Institutional delivery** | **No** | **Yes** |
| Age | 15-19 | 126 (24.15%) | 275 (70.44%) | 68 (15.90%) | 358 (84.10%) | 353 (82.96%) | 73 (17.04%) |
|  | 20-24 | 307 (24.15%) | 1019(75.85%) | 208 (15.49%) | 1,136 (84.51%) | 1,110 (82.58%) | 234 (17.42%) |
|  | 25-29 | 320 (24.38%) | 995 (75.62%) | 190 (14.42%) | 1,127 (85.58%) | 1,087 (82.62%) | 229 (17.38%) |
|  | 30-34 | 279 (27.87%) | 7678 (71.05%) | 171 (17.78) | 792 (82.22%) | 818 (84.95%) | 145  (15.05%) |
|  | 35-39 | 212 (30.00%) | 498 (70.00%) | 146 (20.74%) | 560 (79.26%) | 576 (81.52%) | 130 (18.48%) |
|  | 40-44 | 109 (31.43%) | 239 (68.57%) | 54 (15.54%) | 294 (84.46%) | 283 (81.38%) | 65 (18.62%) |
|  | 45-49 | 31 (45.64%) | 47 (45.64%) | 19 (26.89%) | 51 (73.11%) | 55 (80.51%) | 14 (19.49%) |
| Marital status | Married | 1133 (26.77%) | 3100 (73.23%) | 730 (17.25%) | 3,503 (82.75%) | 3,512 (82.97%) | 721 (17.03%) |
|  | Unmarried | 270 (40.62%) | 669 (71.38%) | 126 (13.42%) | 813 (86.57%) | 771 (82.73%) | 168 (17.92%) |
| Wealth index | Poorest | 439 (40.62%) | 642 (59.38%) | 370 (34.26%) | 711 (65.74% | 928 (85.90%) | 152 (14.10%) |
|  | Poorer | 337 (33.58%) | 667 (66.42%) | 222 (22.11%) | 782 (77.89%) | 824 (82.11%) | 180 (17.89%) |
|  | Middle | 303 (29.98%) | 708 (70.02%) | 138 (13.69%) | 873 (86.31%) | 826 (81.64%) | 186 (18.36%) |
|  | Richer | 213 (19.68%) | 869 (80.32%) | 103 (9.47%) | 980 (90.53%) | 893 (82.46%) | 190 (17.54%) |
|  | Richest | 111 (12.24%) | 883 (88.86%) | 23 (2.29%) | 971 (97.71%) | 812 (81.72%) | 182 (18.59%) |
| Educational status | Uneducated | 370 (36.87%) | 633 (63.13% ) | 321 (32.06 %) | 681 (67.94%) | 863 (86.10%) | 139 (13.90%) |
|  | Primary | 832 (28.81%) | 2056 (71.19%) | 475 (16.44% | 2413 (83.56%) | 2378 (82.34%) | 510 (17.66%) |
|  | Secondary and higher | 202 (15.72%) | 1,081 (84.28%) | 60 (4.68%) | 1,222 (95.32%) | 1,042 (81.29%) | 240 (18.71%) |
| Parity | 1-2 | 480 (20.90%) | 1,816 (79.10%) | 233 (10.15%) | 2063 (89.85%) | 1,897 (82.60%) | 400 (17.40%) |
|  | 3-4 | 431 (26.92%) | 1,171 (73.08%) | 265 (16.57%) | 1,336 (83.43%) | 1,349 (84.23%) | 253 (15.77%) |
|  | ≥5 | 492 (38.60%) | 782 (61.40%) | 358 (28.06%) | 917 (71.94%) | 1,038 (81.40%) | 237 (18.60%) |
| Age at first birth | <18 | 695(30.50%) | 1,584 (69.50%) | 502 (22.04%) | 1,777 (77.96%) | 1,900 (83.39%) | 379 (16.61%) |
|  | ≥18 | 708 (24.47%) | 2,186 (75.53%) | 354 (12.23%) | 2,540 (87.77 %) | 2,383 (82.35%) | 511 (17.65%) |
| Media exposure | No | 793 (32.21%) | 1,669 (67.79%) | 550 (22.62%) | 1,912 (77.67%) | 2,103 (85.44%) | 359 (14.56%) |
|  | Yes | 571 (22.49%) | 1,966 (77.51%) | 281 (11.07%) | 2,255 (88.93%) | 2,036 (80.29%) | 499 (19.71%) |
| Internet use | Never | 1,341  (29.63%) | 3,187 (70.37%) | 849 (18.75%) | 3,679 (81.25 %) | 3,785 (83.59%) | 743 (16.41%) |
|  | In the last 12 months | 51 (9.12%) | 504 (90.88%) | 5 (0.98%) | 550 (99.02%) | 428 (77.13%) | 127 (22.87%) |
|  | Before 12 months | 11 (11.96%) | 78 (88.04%) | 87 (98.33%) | 2 (1.67%) | 70 (78.68%) | 19 (21.32%) |
| Health insurance | No | 1,379 (28.04%) | 3,539 (71.96%) | 838 (17.05%) | 4,079 (82.95%) | 4,087 (83.11%) | 831 (16.89%) |
|  | Yes | 24 (9.54%) | 230 (90.46%) | 18 (6.95%) | 237 (93.05%) | 196 (77.06%) | 58 (22.94%) |
| **Community level characteristics** | | | | | | | |
| Place of residence | Urban | 251 (16.85%) | 1,236 (83.15%) | 70 (4.68%) | 1,417 (95.32%) | 1,213 (82.97%) | 249 (17.03%) |
|  | Rural | 1,153 (31.27%) | 2,532 (68.73%) | 787 (21.34%) | 2,899 (78.66%) | 3,054 (82.87%) | 632 (17.13%) |
| Distance to health facilities | Not big problem | 756 (21.86%) | 2704 (78.14%) | 354 (10.23%) | 3,106 (89.77%) | 2,863 (82.75%) | 597 (17.25 %) |
|  | Big problem | 647 (37.77%) | 1066 (62.23%) | 502 (29.32%) | 1,210 (70.68%) | 1,420 (82.93%) | 292 (17.07%) |
| Community illiteracy | Low illiteracy | 416 (19.18%) | 1,754 (80.82%) | 154 (7.10%) | 2,016 (92.90 %) | 1,758 (81.04%) | 411 (18.96%) |
|  | High illiteracy | 987 (32.87%) | 2016 (67.13%) | 702 (23.38%) | 2,301 (76.62%) | 2,525 (84.09%) | 478 (15.91%) |
| Community wealth index | Low poverty | 388 (17.42%) | 1,839 (82.58%) | 144 (6.45%) | 2,084 (93.55%) | 1,815 (81.48%) | 413 (18.52 %) |
|  | High poverty | 1015 (34.47%) | 1,930 (65.53%) | 713 (24.19%) | 2,233 (75.81%) | 2,468 (83.81%) | 477 (16.19 %) |
| Community media exposure | Low media exposure | 863 (30.84%) | 1,936 (68.62%) | 624 (22.28%) | 2,176 (77.72%) | 2368 (85.57%) | 432 (15.43 %) |
|  | High media exposure | 540 (22.75%) | 1,833 (77.25%) | 232 (9.79%) | 2,141 (90.21%) | 1,916 (80.73%) | 457 (19.27%) |
