# Supplementary material for: Multilevel analysis of dropout from maternal continuum of care and its associated factors: Evidence from 2022 Tanzania Demographic and Health Survey
Source: PLoS One. 2024 May 7;19(5):e0302966. doi: 10.1371/journal.pone.0302966 (PMC11075823; doi:10.1371/journal.pone.0302966)
Supplement: S2 Table — (DOCX) [file pone.0302966.s002.docx]

S2 Table. Random effect result and model comparison for the three maternal CoC

| **Measure of variation for dropout from ANC** | | | | |
| --- | --- | --- | --- | --- |
|  | **Null model** | **Model 1** | **Model 2** | **Model 3** |
| Community level variance (95% CI) | 0.68(0.52, 0.88) | 0.43(0.31, 0.62) | 0.24(0.15, 0.38) | 0.29 (0.19, 0.45) |
| p-value | <0.0001 | <0.0001 | <0.0001 | <0.0001 |
| Deviance | 5776.52 | 4,894.94 | 5,565.72 | 4,827.76 |
| ICC % | 17.12% | 11.7% | 6.67% | 8.70% |
| MOR | 2.19 | 1.86 | 1.60 | 1.67 |
| **Measure of variation for dropout from institutional delivery** | | | | |
| Community level variance (95% CI) | 3.06(2.41, 3.89) | 1.69(1.29, 2.22) | 1.59(1.21, 2.09) | 1.39(1.03, 1.86) |
| p-value | <0.0001 | <0.0001 | <0.0001 | <0.0001 |
| Deviance | 3,850.16 | 3,458.82 | 3,655.28 | 3,383.11 |
| ICC % | 48.17% | 33.92% | 32.57% | 29.66% |
| MOR | 5.27 | 3.44 | 3.25 |  |
| **Measure of variation for dropout from PNC** | | | | |
| Community level variance (95% CI) | 0.65(0.46, 0.92) | 0.61(0.28, 0.88) | 0.56(0.40, 0.81) | 0.54(0.37, 0.79) |
| p-value | <0.0001 | <0.0001 | <0.0001 | <0.0001 |
| Deviance | 4,357.72 | 4,155.77 | 4,321.78 | 4,120.01 |
| ICC % | 16.54% | 15.75% | 14.61% | 14.14% |
| MOR | 2.14 | 2.10 | 2.04 | 2.01 |
