# Supplementary material for: Multilevel analysis of dropout from maternal continuum of care and its associated factors: Evidence from 2022 Tanzania Demographic and Health Survey
Source: PLoS One. 2024 May 7;19(5):e0302966. doi: 10.1371/journal.pone.0302966 (PMC11075823; doi:10.1371/journal.pone.0302966)
Supplement: S3 Table — (DOCX) [file pone.0302966.s003.docx]

S3 Table. Multilevel analysis of factors associated with dropout from the three maternal CoC among Tanzania reproductive-age women.

|  |  |  | **Dropout from** |  |
| --- | --- | --- | --- | --- |
| **Variable name** | **Categories** | **ANC** | **Institutional delivery** | **Postnatal care visit** |
|  |  | **AOR (95% CI)** | **AOR (95% CI)** | **AOR (95% CI)** |
| Age | 15-19 | Reff | Reff | Reff |
|  | 20-24 | 1.16 (0.79, 1.70) | 0.89(0.59, 1.33) | 1.11(0.78,1.58) |
|  | 25-29 | 1.75 (1.01, 3.04)* | 0.65(0.40, 1.06) | 1.19(0.80, 1.76) |
|  | 30-34 | 2.40 (1.10, 5.20)* | 0.62(0.36, 1.07) | 1.39(0.89, 2.19) |
|  | 35-39 | 3.67(1.32,10.15)* | 0.61(0.34, 1.010) | 1.33(0.81, 2.20) |
|  | 40-44 | 5.37(1.47, 18.03)* | 0.41(0.21, 0.78) * | 1.41(0.81, 2.47) |
|  | 45-49 | 6.79 (1.45, 31.94)* | 0.51(0.22, 1.12) | 1.30(0.58, 2.89) |
| Marital status | Married | Reff | Reff | Reff |
|  | Unmarried | 1.37(1.12, 1.68) * | 1.07(0.78, 1.44) | 0.95(0.75, 1.21) |
| Wealth index | Poorest | Reff | Reff | Reff |
|  | Poorer | 0.75(0.61, 0.93) * | 0.76(0.57, 0.97) * | 0.92(0.69, 1.25) |
|  | Middle | 0.62 (0.49,0.79) * | 0.49(0.36, 0.68) * | 1.15(0.84, 1.58) |
|  | Richer | 0.49 (0.36, 0.67) * | 0.50(0.33, 0.76) * | 1.36(0.93, 1.20) |
|  | Richest | 0.32 (0.24, 0.48) * | 0.26(0.13, 0.47) * | 1.67(1.04, 2.68) |
| Educational status | Uneducated | Reff | Reff | Reff |
|  | Primary | 0.99(0.81, 1.20) | 0.80(0.63, 1.01) | 0.80(0.62, 1.03) |
|  | Secondary and higher | 0.94(0.72, 1.24) | 0.55(0.38, 0.79) * | 0.94(0.68, 1.30) |
| Sex of household head | Male | Reff | Reff | Reff |
|  | Female | 0.91(0.75,1.11) | 1.20(0.93, 1.56) | 0.99(0.80, 1.25) |
| Parity | 1-2 | Reff | Reff | Reff |
|  | 3-4 | 1.44(1.16,1.80) * | 2.17(1.58, 2.99) * | 1.08(0.84, 1.39) |
|  | ≥5 | 1.78(1.33, 2.40) * | 3.35(2.22, 5.08) * | 0.85(0.59, 1.21) |
| Age at first birth | <18 | Reff | Reff | Reff |
|  | ≥18 | 1.08(0.92, 1.27) | 0.89(0.71, 1.12) | 0.95(0.78, 1.16) |
| Media exposure | No | Reff | Reff | Reff |
|  | Yes | 1.13(0.96, 1.34) | 0.96(0.76, 1.20) | 0.76(0.61,0.93) * |
| Internet use | Never | Reff | Reff | Reff |
|  | In the last 12 months | 0.54(0.38, 0.76) * | 0.41(0.20, 0.85) * | 0.68(0.50, 0.91) * |
|  | Before 12 months | 0.98 (0.52, 1.84) | 0.56(0.15, 2.06) | 0.65(0.35, 1.18) |
| Health insurance | No | Reff | Reff | Reff |
|  | Yes | 0.53(0.33, 0.84) * | 1.08(0.57, 2.04) | 0.65(0.44, 0.96)* |
| Community level factors |  |  |  |  |
|  |  |  |  |  |
| Place of residence | Urban | Reff | Reff | Reff |
|  | Rural | 0.74(0.57, 0.96) * | 1.34(0.83, 2.16) | 1.01(0.74, 1.38) |
|  |  |  |  |  |
| Region | Central Zone | Reff | Reff | Reff |
|  | Western Zone | 0.71(0.47, 1.06) | 0.52 (0.27, 1.02) * | 0.52 (0.32, 0.87) * |
|  | Northern Zone | 0.48(0.32, 0.73) * | 1.46 (0.78, 2.74) | 0.36 (0.22, 0.57) * |
|  | Southern highland Zone | 0.67(0.43, 1.03) | 0.04 (0.01, 0.16) * | 0.21(0.14, 0.34) * |
|  | Southern Zone | 0.53(0.32, 0.86) * | 0.15(0.06, 0.39) * | 0.51(0.29, 0.67) * |
|  | Southwest highland zone | 0.63(0.43, 0.93) * | 1.09(0.59, 2.03) | 0.82 (0.51, 1.33) |
|  | Lake zone | 0.88(0.63, 1.22) | 1.36(0.81, 2.31) | 0.44(0.29, 0.67) * |
|  | Eastern Zone | 0.40(0.26, 0.61) * | 0.41(0.20, 0.83) * | 0.82(0.51, 1.33) |
|  | Zanzibar | 0.63(0.44, 0.88) * | 1.12 (0.65, 1.94) | 1.08(0.69, 1.41) |
| Distance to health facilities | Not big problem | Reff | Reff | Reff |
|  | Big problem | 1.32(1.12, 1.55) * | 1.47(1.18, 1.83) * | 0.96(0.78, 1.18) |
| Community illiteracy | Low illiteracy | Reff | Reff | Reff |
|  | High illiteracy | 1.27(1.02, 1.56) * | 1.41(0.98, 2.02) | 1.21(0.94, 1.56) |
| Community wealth index | Low poverty | Reff | Reff | Reff |
|  | High poverty | 1.06(0.80,1.40) | 1.42(0.13, 0.90) | 1.08(0.76, 1.52) |
| Community media exposure | Low media exposure | Reff | Reff | Reff |
|  | High media exposure | 1.15(0.93, 1.43) | 0.79(0.18, 0.54) | 0.83(0.63, 1.09) |

Reff indicates the reverence group
