# Supplementary material for: Multilevel analysis of dropout from maternal continuum of care and its associated factors: Evidence from 2022 Tanzania Demographic and Health Survey
Source: PLoS One. 2024 May 7;19(5):e0302966. doi: 10.1371/journal.pone.0302966 (PMC11075823; doi:10.1371/journal.pone.0302966)
Supplement: Attachment — Submitted filename: Response to Reviewer.docx [file pone.0302966.s004.docx]

**Response to Reviewers**

**Manuscript title:** Multilevel analysis of dropout from maternal continuum of care and its associated factors in Tanzania: Evidence from 2022 Tanzania Demographic and Health Survey.

**Manuscript ID:**   PONE-D-23-42201

**Dear editor,**

PLOS ONE

We greatly value the valuable feedback provided by the reviewers. The constructive comments and suggestions from both the reviewers and editors offer significant opportunities to enhance the quality and clarity of the manuscript. We have carefully reviewed each comment and suggestion, and the subsequent pages include a comprehensive response addressing each point raised. Unmarked and marked-up copies of the revised manuscript are uploaded. Moreover, a response that responds to each point raised by the editor/reviewer(s) is highlighted below.

Thank you for the opportunity to improve our manuscript based on your insights.

Sincerely,

Angwach Abrham on behalf of all authors

**Point-by-point response for editor/reviewer comments**

**Response to Reviewer-1**

**1: Introduction**

1. The introduction section should be better structured, and it should be explicitly stated that previous studies have shown that a very small proportion of women receive care throughout the CoC, and that many of them dropout in the CoC and therefore do not receive the completed care package, reducing the scope of health benefits that the CoC offers for the woman and her child.

**Authors’ response:** Thank you reviewer for your comment. We have duly addressed this (pp: 3-5).

2. The introduction should establish more clearly the concept of dropout of the CoC and the different moments in which it can be observed throughout the CoC. Likewise, in the available literature there is not a single definition for CoC dropout. On the contrary, there are restrictive definitions of the different types of dropouts for prenatal care, childbirth and postpartum. It is recommended to refer to it, its strengths, weaknesses, and the factors that have been associated with the results available in the evidence.

**Authors’ response:** Thank you reviewer for your comment. We have duly addressed this (pp 4-5, line 77-86).

**2: Method**

3. In the methodology section, it is suggested to include the way in which the independent variables were analysed at the individual and community levels. Likewise, for the latter, indicate whether they were also extracted with information from the same survey or from other sources of information.

**Authors’ response:** Thank you reviewer for your comment. All variables included in this study were extracted from the same survey (2022 TDHS). All individual-level variables were taken from the survey except media exposure, which was generated by adding exposures to radio and/or television. Community-level variables (i.e. community illiteracy and community wealth index) were generated by aggregating individual-level variables into community-level variables. Other community-level variables (i.e. place of residence, geographic area, and distance to the health facility) were simply taken from the survey as it is. Overall, we have explicitly described these (please see revised manuscript; pp: 7-8, lines 139-147).

**3: Results**

4. In the results section, in Figure 1, the bars for the different moments of the CoC and for the general analysis do not add up to 100% except for the dropout for PNC. It is suggested to review and correct.

**Authors’ response:** Thank you reviewer for your comment. We have corrected it in the revised Figure 1.

**4: Discussion**

5. In the discussion section on lines 252-254 it is noted, “The overall rate of maternal CoC (83.86%) is lower than the rates reported in studies conducted in Ethiopia [19], Tanzania [18], Uganda [21], Gambia, and Ghana [24], and higher than the reports from a study conducted in Kenya [25]” However, in reality, according to Figure 1, this value It is suggested to review and correct. refers to the percentage of women who do not receive the full attention of the CoC continues.

**Authors’ response:** Thank you reviewer for your comment. We have revised and corrected it in the revised manuscript (pp 19, line 276).

6. It is suggested to add in the discussion section a reflection on how the findings of the present study contribute to the evidence that exists in the literature on the CoC linkages and what hypotheses the presented findings may arise in this sense for this country.

**Authors’ response:** Thank you reviewer for your suggestions. We have addressed it in the revised manuscript (pp: 19)
