# Supplementary material for: Multilevel analysis of dropout from maternal continuum of care and its associated factors: Evidence from 2022 Tanzania Demographic and Health Survey
Source: PLoS One. 2024 May 7;19(5):e0302966. doi: 10.1371/journal.pone.0302966 (PMC11075823; doi:10.1371/journal.pone.0302966)
Supplement: Attachment — Submitted filename: Response to Reviewers.docx [file pone.0302966.s005.docx]

**Response to Reviewers**

**Manuscript title:** Multilevel analysis of dropout from maternal continuum of care and its associated factors in Tanzania: Evidence from 2022 Tanzania Demographic and Health Survey.

**Manuscript ID:**   PONE-D-23-42201R1

**Dear editor,**

PLOS ONE

We greatly value the valuable feedback provided by the editor and reviewers. These constructive comments and suggestions offer significant opportunities to enhance the quality and clarity of the manuscript. We have carefully reviewed each comment and suggestion, and indicated point-by-point comprehensive responses addressing each point raised on subsequent pages. Moreover, unmarked and marked-up copies of the revised manuscript are uploaded.

Thank you for the opportunity to improve our manuscript based on your insights.

Sincerely,

Angwach Abrham on behalf of all authors

**Point-by-point response for editor/reviewer comments**

**Response to Editor-**

1. Dear Authors, please revise your manuscript in detail and address each and every concern of the reviewers. Mainly, you are expected to clarify the methods used for this study, the description of the results section and the discussion section.

**Authors’ response:** Thank you editor for your suggestions. We have carefully revised these in respective sections.

2. Please correct the country name 'Kenia' as Kenya, line 254 in the discussion section.

**Authors’ response:** Thank you for your comment. This was a typo; we have corrected it.

3. I suggest that discussion section start with a paragraph highlighting the most relevant accomplishments of the research. This section can be improved as follows.

a) Main findings in the first paragraph

b) Comparison with existing literature

c) Strengths and Weaknesses of the study should be clearly highlighted

d) Conclusion

**Authors’ response:** Thank you very much for this suggestion. We have addressed it in the revised manuscript (Discussion section).

**Response to Reviewer-**

**1: Results**

1. Check the paragraph starting @ line 209,

its not clear to start with and the numbers indicating dropouts of ANC are incongruent. 27.13% vs (82.62%).

**Authors’ response:** Thank you reviewer for your careful consideration. This was an editorial issue; we have rephrased it accordingly.

2. Analysis of dropout to ANC, institutional delivery and PNC

If you have to analyze each components of the CoC, then what is the value of merging them to one variable (CoC)? And the same applies to your discussion, you need to have a good justification to have a question of continuum of care dropout and discuss about a single area of care dropout - ANC or other components.

**Authors’ response:** Thank you reviewer for your comments. Incorporation of all these 3 components in the formulation of the CoC variable ensures that essential preventive care services are comprehensively administered during these critical points of contact (Introduction: lines 98-104; Methods: lines 125-127; 135-136; 142-144). Moreover**,** COC outlines important connections in time (when the care is given) and place (where the care is given). To progress toward higher coverage of crucial and evidence-based interventions, women stay healthy, and newborns have the best start in life when they receive high-quality care during pregnancy, delivery, and the early postnatal period. Analyzing each component of the CoC separately is important to identify which component of CoC had high dropout and to minimize the health cost by intervening on the most common dropout. So, we supported the overall CoC findings with each component of the continuum.

**2: Discussion**

1. It is recommended to start a discussion by stating your purpose of the study and then revealing the major findings.

**Authors’ response:** Thank you for your comments. We have addressed this in the revised manuscript (lines 261-266)

2. Your justifications for older mothers nullified your justification for grand multiparity all in a single paragraph. It needs revision, I think.

**Authors’ response:** Thank you so much for your significant insight. We have addressed it in the revised manuscript.

3. 4th paragraph (line 294)

Odds of dropouts of CoC are lower among married women and unmarried women have higher odds of dropout for ANC - your results stated and I am not clear how these statements goes different. They look the same but you stated otherwise.

**Authors’ response:** Thank you very much for your comments and suggestions. We have rephrased it.
